# Supplementary material for: Record ages of non-Markovian scale-invariant random walks
Source: Nat Commun. 2023 Oct 9;14:6288. doi: 10.1038/s41467-023-41945-9 (PMC10562453; doi:10.1038/s41467-023-41945-9)
Supplement: Supplementary file 1 — Supplementary Information [file 41467_2023_41945_MOESM1_ESM.pdf]

SUPPLEMENTARY INFORMATION
Record Ages of Non-Markovian Scale-Invariant Random Walks

L. Régnier, M. Dolgushev, and O. Bénichou

CONTENTS

S1. Scaling theory 1
A. Definitions 1
B. Scale-invariance of the time T_n to break n records and of its increments 1
C. Characteristic exponents of the record age distribution 2
D. General scaling criteria showing that the correlations between record ages are asymptotically irrelevant for their maximum 3
1. Effective independence criteria 3
2. General form of $\mathbb{P}(\tau_k \geq T)$ 4
3. Cross-correlations 4
4. Variance of max 5
5. Conclusion 5

S2. Non-Markovian random walks (RWs) 5
A. Definition of the non-Markovian RW models 5
B. Systematic numerical check of the scale-invariance of the time increments 8
C. Systematic numerical check of the asymptotic independence of record ages 10

S3. Data analysis 12
A. Details on the datasets used in the main text 12
B. Characterization and parametrization of the data used in the main text 12
C. Analysis of complementary datasets 14
D. Datasets displaying aging of the increments 16

Supplementary References 17

S1. SCALING THEORY

We provide here the detailed calculations corresponding to the general scaling theory developed in the main text.

A. Definitions

We assume that the random walk (RW) $(X_t)_{t=0,\ldots}$ converges at large time to a continuous and non-smooth scale-invariant process. Under these conditions, the process is characterized by a walk dimension $d_{\rm w} > 1$, such that $X_t \propto t^{1/d_{\rm w}}$, and the random variable $X_t/t^{1/d_{\rm w}}$ is independent of t. We more generally assume that X_t has scale-invariant increments with a potential ageing, meaning that, for $1 \ll t \ll T$, $X_{t+T} - X_T \propto t^{1/d^0_{\rm w}} T^{\alpha/2}$. This defines the aging exponent α [S1, S2] and an effective walk dimension at short times $d^0_{\rm w} \equiv (d_{\rm w}^{-1} - \alpha/2)^{-1}$.

To describe the properties of the RWs, we will use their continuous limit, which requires to introduce a microscopic cut-off either in time or space (as done in [S3]). Relying here on a spatial cut-off Δx, we consider the time T_{x_0} to first reach the level $x_0 = n\Delta x$ starting from the origin, as the continuous counterpart of the time T_n to break n records.

B. Scale-invariance of the time T_n to break n records and of its increments

First, we check that the temporal scale-invariance of X_t leads to the spatial scale-invariance of T_{x_0}. Indeed, since $X_t/t^{1/d_{\rm w}}$ is asymptotically independent of time t, $X_{T_{x_0}}/T_{x_0}^{1/d_{\rm w}} = x_0/T_{x_0}^{1/d_{\rm w}}$ is asymptotically independent of x_0

($X_{T_{x_0}} = x_0$ because of continuity). Consequently, T_{x_0} is scale-invariant in the sense that $T_{x_0}/x_0^{d_{\rm w}}$ is independent of x_0. Taking $\Delta x = 1$ and $x_0 = n\Delta x = n$ results in the following form for the cumulative distribution:

$$\mathbb{P}(T_n \le T) = \Phi\left(T/n^{d_{\rm w}}\right) \ , \tag{S1}$$

where the scaling function Φ is independent of T and n (see Supplementary Figure 2 for numerical check of the number of records n scale-invariance with time T for representative non-Markovian RWs).

Second, we show that the scale-invariance of the increments $X_{t+T} - X_T$, $X_{t+T} - X_T \propto t^{1/d_{\rm w}-\alpha/2}T^{\alpha/2} = t^{1/d_{\rm w}^0}T^{\alpha/2}$, implies the scale-invariance of $T_{x_0+x_1} - T_{x_0}$. Consider the random variable $\frac{X_{T+t}-X_T}{T^{\alpha/2}t^{1/d_{\rm w}^0}}$, which is independent of t and T as long as $1 \ll t \ll T$. By replacing T by T_{x_0} and t by $T_{x_0+x_1} - T_{x_0}$, we find that

$$\frac{X_{T_{x_0+x_1}} - X_{T_{x_0}}}{T_{x_0}^{\alpha/2}(T_{x_0+x_1} - T_{x_0})^{1/d_{\rm w}^0}} = \frac{x_1}{T_{x_0}^{\alpha/2}(T_{x_0+x_1} - T_{x_0})^{1/d_{\rm w}^0}},$$

is asymptotically independent of x_0 and x_1 for $1 \ll x_1 \ll x_0$. Using that $T_{x_0}/x_0^{d_{\rm w}}$ is independent of x_0, we finally obtain that

$$\frac{T_{x_0+x_1} - T_{x_0}}{(x_1/x_0^{d_{\rm w}\alpha/2})^{d_{\rm w}^0}} = \frac{T_{x_0+x_1} - T_{x_0}}{x_1^{d_{\rm w}^0}x_0^{d_{\rm w}-d_{\rm w}^0}}$$

is independent of x_0 and x_1. In other words, we have the scale invariance of $T_{x_0+x_1} - T_{x_0}$, $T_{x_0+x_1} - T_{x_0} \propto x_1^{d_{\rm w}^0}x_0^{d_{\rm w}-d_{\rm w}^0}$. Taking $\Delta x = 1$, $x_0 = m\Delta x$ and $x_1 = n\Delta x$ implies the scaling $T_{m+n} - T_m \propto n^{d_{\rm w}^0}m^{d_{\rm w}-d_{\rm w}^0}$. It means that the cumulative distribution in the limit $1 \ll n \ll m$ and $1 \ll T \ll m^{d_{\rm w}}$ can be written as

$$\mathbb{P}(T_{m+n} - T_m \le T) = \Psi\left(\frac{T}{n^{d_{\rm w}^0}m^{d_{\rm w}-d_{\rm w}^0}}\right), \tag{S2}$$

where the scaling function Ψ is independent of T, n, and m. This scale-invariance of the time increments is systematically checked numerically for a number of representative non-Markovian RWs in Supplementary Figure 3.

C. Characteristic exponents of the record age distribution

We derive the exponents governing the algebraic decay of the record age distribution $S(n,\tau)$ by elaborating the arguments sketched in the main text.

We note by τ_k the $k^{\rm th}$ record age or, in the continuum setting, the time to reach level $(k+1)\Delta x$ starting from the first arrival at level $k\Delta x$. T_n being the time to first reach level $n\Delta x$, it is given by the sum of the $\{\tau_k\}$,

$$T_n = \sum_{k=0}^{n-1} \tau_k \ . \tag{S3}$$

We make the self-consistent assumption that the tail distribution of τ_k is algebraic of exponent smaller than 1. As a consequence, the sum (S3) is controlled by the largest τ_k [S4], i.e.

$$\sum_{k=0}^{n-1} \tau_k \sim \max\left(\tau_0, \dots, \tau_{n-1}\right) \ . \tag{S4}$$

We now assume (see the next subsection for an analytical justification, as well as Supplementary Figure 4 and Supplementary Figure 5 for systematic numerical checks for a number of representative non-Markovian RWs) that the record ages τ_k involved in Eq. (S4) are asymptotically ($n \gg 1$) effectively independent, which leads to

$$\mathbb{P}(T_n \le T) \simeq \prod_{k=0}^{n-1} \mathbb{P}(\tau_k \le T) = \prod_{k=0}^{n-1}(1 - S(k,T)). \tag{S5}$$

We search now the exponents y_i and ϵ_i characterizing the tail distribution of τ_k, $S(k,T) \propto k^{-1+\epsilon_1}T^{-y_1}$ for $T \ll k^{d_{\rm w}}$ (regime 1) and $S(k,T) \propto k^{-1+\epsilon_2}T^{-y_2}$ for $T \gg k^{d_{\rm w}}$ (regime 2), see Eq. (1) of the main text.

We start with $T \gg n^{d_\mathrm{w}}$, so that all $S(k,T)$ are in the same regime $T \gg k^{d_\mathrm{w}}$. Using Eq. (S5),

$$\mathbb{P}(T_n \leq T) \simeq \exp\left[-\text{cste.}\sum_{k=1}^{n-1}\frac{1}{k^{1-\epsilon_2}}\frac{1}{T^{y_2}}\right] \simeq \exp\left[-\text{cste.}\frac{n^{\epsilon_2}}{T^{y_2}}\right] \tag{S6}$$

The scale-invariance of T_n (Eq. (S1)) implies that $\epsilon_2/y_2 = d_\mathrm{w}$.

Next, for $T \ll n^{d_\mathrm{w}}$, $S(n,T) \propto n^{-1+\epsilon_1}T^{-y_1}$. By splitting the sum over k between the regions $k^{d_\mathrm{w}} \ll T$ and $k^{d_\mathrm{w}} \gg T$, we have

$$\begin{aligned}\mathbb{P}(T_n \leq T) \simeq &\exp\left[-\text{cste.}\sum_{k=1}^{T^{1/d_\mathrm{w}}}\frac{1}{k^{1-\epsilon_2}}\frac{1}{T^{y_2}} - \text{cste.}\sum_{k=T^{1/d_\mathrm{w}}}^{n-1}\frac{1}{k^{1-\epsilon_1}}\frac{1}{T^{y_1}}\right]\\ &\propto \exp\left[-\text{cste.}\frac{n^{\epsilon_1}}{T^{y_1}}\right] = \exp\left[-\text{cste.}\left(\frac{n^{\epsilon_1/y_1}}{T}\right)^{y_1}\right].\end{aligned} \tag{S7}$$

This result, together with Eq. (S1), yields $\epsilon_1/y_1 = d_\mathrm{w} = \epsilon_2/y_2$. In particular, the survival probability of the record-ages admits a scaling form in T and k:

$$S(k,T) = \frac{1}{k}\psi(T/k^{d_\mathrm{w}})\ . \tag{S8}$$

To proceed further, we look at the cumulative distribution of the increments $T_{m+n} - T_m$ in the limit $1 \ll n \ll m$ and $1 \ll T \ll m^{d_\mathrm{w}}$,

$$\begin{aligned}\mathbb{P}(T_{m+n} - T_m \leq T) &= \mathbb{P}\left(\sum_{k=m}^{n+m-1}\tau_k \leq T\right)\\ &\simeq \exp\left[-\text{cste.}\sum_{k=m}^{n+m-1}\frac{1}{k^{1-\epsilon_1}}\frac{1}{T^{y_1}}\right] \simeq \exp\left[-\text{cste.}\frac{n}{m^{1-\epsilon_1}T^{y_1}}\right] = \exp\left[-\text{cste.}\left(\frac{n^{1/y_1}}{m^{(1-\epsilon_1)/y_1}T}\right)^{y_1}\right]\ .\end{aligned} \tag{S9}$$

Using finally Eq. (S2), we obtain $y_1 = 1/d_\mathrm{w}^0$ and $\frac{1-\epsilon_1}{y_1} = d_\mathrm{w}^0 - d_\mathrm{w}$, leading to $\epsilon_1 = d_\mathrm{w}/d_\mathrm{w}^0$. This provides the exponents y_1 and ϵ_1 of regime 1 ($\tau_n \ll n^{d_\mathrm{w}}$). We note that the algebraic decay of $S(n,\tau)$ holds after the time τ_n such that

$$X_{\tau_n+T_n} - X_{T_n} \propto T_n^{\alpha/2}\tau_n^{1/d_\mathrm{w}^0} \propto (n\Delta x)^{d_\mathrm{w}\alpha/2}\tau_n^{1/d_\mathrm{w}^0} = (n\Delta x)^{1-d_\mathrm{w}/d_\mathrm{w}^0}\tau_n^{1/d_\mathrm{w}^0} \gg \Delta x\ . \tag{S10}$$

This gives the lower bound $\tau_n \gg n^{d_\mathrm{w}-d_\mathrm{w}^0}$ in Eq. (1) of the main text for regime 1.

For times $\tau_n \gg n^{d_\mathrm{w}}$ (regime 2), the record age τ_n is much larger than the typical time needed to break all the previous records. At this time scale, the memory of the n broken records no longer affects the algebraic time decay of $S(n,\tau)$, which is thus given by the usual persistence exponent $\theta = y_2$ (defined as $\mathbb{P}(T \geq \tau) \propto \tau^{-\theta}$ where T is the time needed to reach a given value for the first time). Knowing that $\epsilon_2/y_2 = d_\mathrm{w}$, we obtain $\epsilon_2 = d_\mathrm{w}\theta$.

By combining the results derived in this section, we finally obtain Eq. (1) of the main text.

D. General scaling criteria showing that the correlations between record ages are asymptotically irrelevant for their maximum

1. *Effective independence criteria*

Here we show that in the calculation of the distribution of the maximum of the record ages, $M_n \equiv \max(\tau_0,\ldots,\tau_{n-1})$, the random variables τ_k can be treated as effectively independent. To do so, we extend the criteria of Ref. [S5]. Dividing the set $(\tau_0,\ldots,\tau_{n-1})$ in two subsets, $(\tau_0,\ldots,\tau_{n/2-1})$ and $(\tau_{n/2},\ldots,\tau_n)$, this criteria states that the correlations between the τ_k are irrelevant if (a) the typical mean cross-correlation between the subsystems is much smaller than (b) the variance of the maximum of record ages of the whole system.

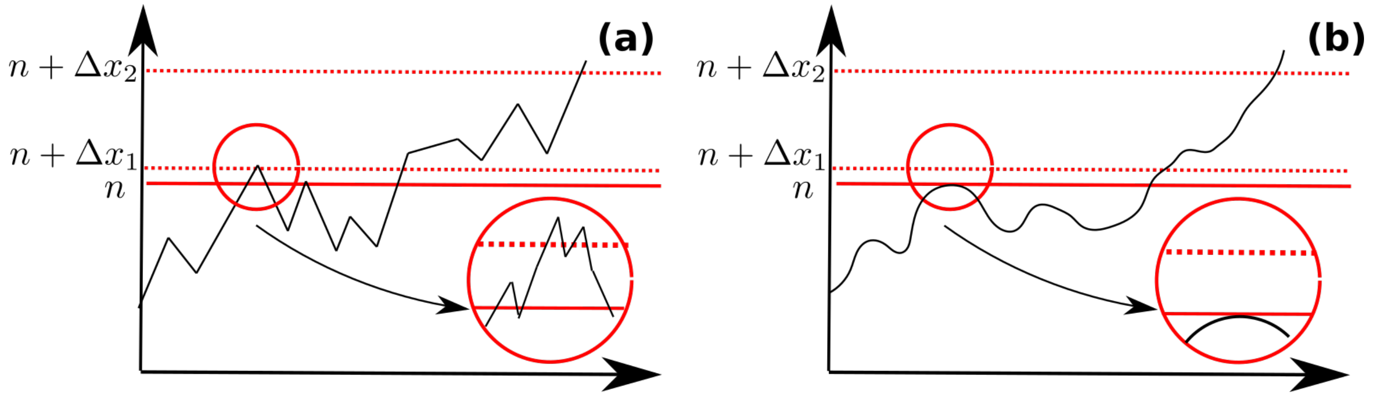

Supplementary Figure 1. **Non-smoothness versus smoothness.** We compare **(a)** a non-smooth process to **(b)** a smooth process by zooming on the trajectory that reaches level n for the first time. For the non-smooth process the time needed to cross level $n+\Delta x_1$ (with a $\Delta x_1 \ll n$ small enough) starting from level n is significantly smaller than for the smooth process.

Here, however, the random variables $\{\tau_k\}$ have diverging second moments, see Eq. (1) of the main text. To overcome this difficulty, we extend the criteria of Ref. [S5] which uses variances of random variables and consider fractional moments of $\{\tau_k\}$ that converge, see below. Also, the original work of Ref. [S5] considers random variables that are Gaussian and identically distributed. Here we extend the approach to the distribution given in Eq. (1) of the main text.

2. *General form of* $\mathbb{P}(\tau_k \geq T)$

We use dimensional analysis to obtain the scaling form of the tail distribution of record ages, without assuming their independence. There are no additional hypotheses on the RW processes beyond those already requested:
(i) continuity, (ii) non-smoothness, and (iii) asymptotic scale-invariance.

We recall that the probability to reach level $(k+1)\Delta x = n + \Delta x$ starting from $k\Delta x = n$ at a time larger than T is given by a function of microscopic cut-off Δx (length), the level number n (length) and T (time). Based on dimensional analysis and scale-invariance of the process, the tail distribution of the record ages takes the functional form $\mathbb{P}(\tau_k \geq T) = F(\Delta x/n, T/n^{d_\mathrm{w}})$. We are interested in the limit $\Delta x/n \ll 1$ and $T \sim n^{d_\mathrm{w}}$, and consider (without restriction of generality) the limit behaviour

$$\mathbb{P}(\tau_k \geq T) = F\left(\frac{\Delta x}{n}, \frac{T}{n^{d_\mathrm{w}}}\right) \sim \left(\frac{\Delta x}{n}\right)^{\beta} \psi(T/n^{d_\mathrm{w}}) \tag{S11}$$

with β an exponent that we determine in the following.

For continuous non-smooth processes, when reaching the nth level, the RW crosses the level n infinitely often [S6], see Supplementary Figure 1**a** for an illustration. In particular, this means that when Δx is small compared to n, there is a time, much smaller than $T \sim n^{d_\mathrm{w}}$, at which the trajectory crosses level $n + \Delta x$. Thus, the probability to reach level $n + \Delta x$ at a time larger than T goes to 0 when Δx goes to 0, which shows that the exponent β in Eq. (S11) is strictly positive, $\beta > 0$.

3. *Cross-correlations*

We wish to find an upper bound of the typical correlations between different τ_k of the set $(\tau_0, \dots, \tau_n)$, for example, typically quantified by the correlation between $\tau_{n/4}$ and $\tau_{3n/4}$. However, computing directly their covariance $\mathrm{Cov}\left(\tau_{n/4}, \tau_{3n/4}\right)$ would lead to a diverging result, because of a heavy-tailed τ_k distribution. Therefore, we will make use of the fractional powers of record ages, $\{\tau_k^q\}$, where the value of q allows converging second moments of these new variables.

An upper bound of the covariance $\mathrm{Cov}\left(\tau_{n/4}^q, \tau_{3n/4}^q\right)$ is obtained by using the Cauchy-Schwarz inequality

$$\mathrm{Cov}\left(\tau_{n/4}^q, \tau_{3n/4}^q\right) \leq \sqrt{\mathrm{Var}(\tau_{n/4}^q)\mathrm{Var}(\tau_{3n/4}^q)}. \tag{S12}$$

The scaling behavior of each of the variances can be found based on Eq. (S11) for continuous non-smooth scale-invariant processes, leading to the moments ($k = 1, 2$)

$$\langle \tau_n^{kq} \rangle \propto n^{-\beta} \int_1^{\infty} \mathrm{d}\tau \, \tau^{qk-1} \psi(\tau/n^{d_\mathrm{w}}) \propto n^{-\beta + qkd_\mathrm{w}}. \tag{S13}$$

Note that for $\beta > 0$, $\langle \tau_n^{2q} \rangle$ dominates $\langle \tau_n^q \rangle^2$, so that $\mathrm{Var}(\tau_n^q) \lesssim \langle \tau_n^{2q} \rangle$. Using this, we get the following final estimation of the upper bound for the typical cross-correlations:

$$\mathrm{Cov}\left(\tau_{n/4}^q, \tau_{3n/4}^q\right) \leq \sqrt{\mathrm{Var}(\tau_{n/4}^q)\mathrm{Var}(\tau_{3n/4}^q)} \propto n^{-\beta + 2qd_\mathrm{w}}. \tag{S14}$$

This last inequality finally provides an upper bound of the typical correlations between the subsets $(\tau_0, \ldots, \tau_{n/2-1})$ and $(\tau_{n/2}, \ldots, \tau_n)$.

4. Variance of max

The fluctuations of the maximum of the record ages $M_n \equiv \max(\tau_0, \ldots, \tau_{n-1})$ are estimated by relying on the variance of $M_n^q \equiv \max(\tau_0^q, \ldots, \tau_{n-1}^q)$. Based on Eqs. (S1) and (S3) and on the self-consistently checked Eq. (S4), the maximum's moments ($k = 1, 2$) can be written as:

$$\langle M_n^{kq} \rangle \sim \int_1^{\infty} \mathrm{d}M \, M^{qk-1} (1 - \Phi(M/n^{d_\mathrm{w}})) \propto n^{qkd_\mathrm{w}}. \tag{S15}$$

We choose q such that the integral in Eq. (S15) converges. Because M_n is scale invariant (M_n/n^{d_w} is a non-deterministic n independent random variable), we finally have

$$\mathrm{Var}\left(M_n^q\right) \propto \langle M_n^{2q} \rangle \propto n^{2qd_\mathrm{w}}. \tag{S16}$$

5. Conclusion

We now can compare the typical cross-correlations to the fluctuations of the maximum,

$$\mathrm{Cov}\left(\tau_{n/4}^q, \tau_{3n/4}^q\right) \leq \sqrt{\mathrm{Var}(\tau_{n/4}^q)\mathrm{Var}(\tau_{3n/4}^q)} \propto n^{-\beta + 2qd_\mathrm{w}} \ll n^{2qd_\mathrm{w}} \propto \mathrm{Var}\left(M_n^q\right). \tag{S17}$$

We conclude that the fluctuations of the maximum of record ages dominate the correlations between two record ages, so that the random variables τ_k can be considered as effectively independent.

We note that, a priori, the correlations between $\{\tau_k\}$ are not negligible if one of the three hypotheses ((i) continuity, (ii) non-smoothness, and (iii) scale-invariance) breaks. While the absence of scale-invariance and of continuity would immediately invalidate calculations of this section, it is not evident for smooth processes. The necessity of the assumption of non smoothness originates from the following argument. When a realisation of a smooth process just reaches the level n, in the following the trajectory can go back to position $x < n$ without crossing level n, see Supplementary Figure 1**b**. Thus, for smooth processes, the probability for a RW trajectory to reach level $n + \Delta x$ at a time T for Δx arbitrary small is finite, implying $\beta = 0$ in Eq. (S11), which invalidates the estimations after it.

S2. NON-MARKOVIAN RANDOM WALKS (RWS)

A. Definition of the non-Markovian RW models

In this subsection, we present the non-Markovian random walk (RW) processes, which are used in Fig. 2 of the main text. These processes encompass the three classes of different statistical statistical mechanisms giving rise to a non-Markovian evolution discussed in the main text and depicted in Fig. 1 of the main text. Supplementary Table 1 provides a summary of their characteristic parameters, namely d_w, α, d_w^0 and θ.

Model	$d_{\rm w}$	α	$1/d_{\rm w}^0$	θ
fBm	$1/H$	0	H	$1-H$
qfBm	$1/H$	0	H	$\theta(H)$ (see [S7])
eRW	2	0	$1/2$	$3/2-2\beta$
SATW	2	0	$1/2$	$e^{-\beta}/2$
SESRW	$\frac{2+\kappa}{1+\kappa}$	0	$\frac{2+\kappa}{1+\kappa}$	≈ 0.3
TSAW	$3/2$	0	$3/2$	$1/3$
subALL	$3-a$	$\frac{a-1}{3-a}$	$1/2$	$(2-a)/(3-a)$
supALL	$1+a$	$\frac{1-a}{1+a}$	$1/2$	$a/(1+a)$
sBm	$2/\beta$	$\beta-1$	$1/2$	$\beta/2$

Supplementary Table 1. Summary of the non-Markovian models considered in this study and of their characteristic parameters.

(a) *Fractional Brownian motion (fBm).* The fBm is a non-Markovian Gaussian process, with stationary increments. Thus, an fBm X_t of Hurst index H is defined by its covariance

$$\mathrm{Cov}\left(X_t, X_{t'}\right) = \frac{1}{2}\left(t^{2H} + t'^{2H} - |t-t'|^{2H}\right) . \tag{S18}$$

The steps $\eta_t = X_t - X_{t-1}$ are called fractional Gaussian noise (fGn). Nowadays, the fBm is broadly spread and its implementations could be found in standard packages of python or Wolfram Mathematica. Besides, the survival probability of fBm is characterized by the persistence exponent $\theta = 1-H$, which was derived in [S8–S10].

(b) *Quenched fBm (qfBm).* This process is an extension of fBm to quenched initial conditions, which results in non-stationary increment statistics. In particular, it describes the height fluctuations under Gaussian noise of an initially flat interface. Then X_t corresponds to the height of the interface at position $x=0$, $X_t = h(0,t)$, $h(x,t)$ following the Stochastic Differential Equation (SDE)

$$\partial_t h(x,t) = -\left(-\Delta\right)^{z/2} h(x,t) + \eta(x,t). \tag{S19}$$

Here $\eta(x,t)$ is a Gaussian noise with possible spatial correlations. We solve numerically this SDE with a spatial discretization $\Delta x = 1$ and a time discretization $\Delta t = 0.1$. The system is initially flat, $h(x,t=0) = h_0$. The model at $z=2$ with space-independent noise is a non-stationary fBm of Hurst exponent $H = (1-1/z)/2 = 1/4$ which corresponds to the continuous limit of a solid-on-solid model [S11]. The persistence exponent θ was numerically estimated to be $\theta = 1.55 \pm 0.02$ [S7].

(c) *Elephant RW (eRW).* This process is representative of interactions with its own trajectory. At time t, the step η_t is drawn uniformly among all the previous steps η_i $(i<t)$ and is reversed with probability β. The persistence exponent was determined in [S12] to be $\theta = 3/2 - 2\beta$.

(d) *Self-attractive walk (SATW).* This model is a prototypical example of self-interacting RWs. In the SATW model [S12–S15], the RW at position i jumps to a neighbouring site $j = i \pm 1$ with probability depending on the number of times n_j it has visited site j,

$$p(i \to j) = \frac{\exp\left[-\beta H(n_j)\right]}{\exp\left[-\beta H(n_{i-1})\right] + \exp\left[-\beta H(n_{i+1})\right]}, \tag{S20}$$

where $H(0) = 0$, $H(n>0) = 1$ and $\beta > 0$. It was shown in [S12] that the persistence exponent of this RW is given by $e^{-\beta}/2$.

(e-f) *Exponential self-repelling RW.* This is another example of self-interacting RW. In this model, the RW at position i jumps to a neighbouring site $j = i \pm 1$ with probability depending on the number of times n_j it has visited site j,

$$p(i \to j) = \frac{\exp\left[-\beta n_j^\kappa\right]}{\exp\left[-\beta n_{i-1}^\kappa\right] + \exp\left[-\beta n_{i+1}^\kappa\right]} \tag{S21}$$

where κ and β are two positive real numbers. It was shown [S16] that the walk dimension of such walks is given by $d_\mathrm{w} = \frac{1+\kappa}{2+\kappa}$. In the case $\kappa = 1$ (the True Self-Avoiding Walk, TSAW, [S17–S19]), the persistence exponent is given by [S15] $\theta = 1/3$, while for $\kappa < 1$ (the Sub-Exponential Self-repelling Walk, SESRW, [S16, S20]), its numerical estimation is $\theta \approx 0.3$.

(g-h) *Average Lévy Lorentz gas (ALL).* This model is emblematic for RWs with spatially-dependent steps; Its different properties are described in [S21, S22]. We consider a RW on a $1d$ lattice with position dependent reflection or transmission probabilities $r(k)$ or $t(k)$. In the subdiffusive model (subALL), the transmission coefficient is taken to be

$$t(k) = \begin{cases} \frac{a\sin(\pi a)\zeta(1+a)}{2\pi|k|^{1-a}} & \text{if } |k| > 0 \\ 1/2 & \text{otherwise} \end{cases} \tag{S22}$$

In the continuous setting, this is equivalent to a space dependent diffusion coefficient for $0 < a < 1$, $D_a(x) = \left(4\Lambda|x|^{1-a} - 2\right)^{-1}$ where $\Lambda = \frac{\pi}{a\sin(\pi a)\zeta(1+a)}$. The persistent exponent is given by $\theta = 1 - 1/(3+a)$ [S22]. In the superdiffusive model (supALL), the reflection coefficient is taken to be

$$r(k) = \begin{cases} \frac{a\sin(\pi a)\zeta(1+a)}{2\pi|k|^{1-a}} & \text{if } |k| > 0 \\ 1/2 & \text{otherwise.} \end{cases} \tag{S23}$$

In the continuous setting, this is equivalent to a space dependent diffusion coefficient for $0 < a < 1$, $D_a(x) = \Lambda|x|^{1-a} - 1/2$ where $\Lambda = \frac{\pi}{a\sin(\pi a)\zeta(1+a)}$. The persistent exponent is given by $\theta = 1 - 1/(1+a)$ [S22].

(i) *Scaled Brownian motion (sBm).* The sBm model represents RWs with time-dependent steps [S23–S26]. Starting from X_t^0 a process with i.i.d. symmetric jumps η_i^0 with finite variance, we define the scale process of parameter β by $X_t \equiv X^0_{\lfloor t^\beta \rfloor}$, or equivalently $\eta_t = \sum_{k=\lfloor (t-1)^\beta \rfloor}^{\lfloor t^\beta \rfloor - 1} \eta_k^0$. A second way to define the sBm on discrete times is to consider steps η_t following a binomial distribution of parameters $(N_t, 1/2)$ where N_t is Poisson distributed of average $\lambda(t) = t^{\beta-1}$ (the one used in Supplementary Figure 2). In the continuous setting, it amounts to a time-dependent overdamped Langevin equation,

$$\frac{dX_t}{dt} = \sqrt{2D\beta t^{\beta-1}}\eta_t, \tag{S24}$$

where η_t is a white noise of unit variance. The persistence exponent of sBm is $\theta = \beta/2$ [S24].

The sBm model allows one to compute the distribution of $\tau_{x_0} \equiv T_{x_0+\Delta x} - T_{x_0}$ analytically in the continuous limit,

$$\begin{aligned} \mathbb{P}(\tau_{x_0} \geq \tau) &= \int_0^\infty dT_0 \mathbb{P}(T^0_{x_0} = T_0)\mathbb{P}(T^0_{\Delta x} \geq (\tau + T_0^{1/\beta})^\beta - T_0) \\ &= \int_0^\infty dT_0 \frac{x_0}{\sqrt{2\pi T_0^3}} \exp\left[-\frac{x_0^2}{2T_0}\right] \mathrm{erf}\left(\frac{\Delta x\sqrt{2}}{\sqrt{(\tau + T_0^{1/\beta})^\beta - T_0}}\right) \end{aligned} \tag{S25}$$

We plot this exact formula in Fig. 2 of the main text ($x_0 = n$, $\Delta x = 1$) and in Supplementary Figure 3 ($x_0 = m$, $\Delta x = n$). In the case of small Δx,

$$\mathbb{P}(\tau_{x_0} \geq \tau) \sim \frac{2\Delta x}{\pi} \int_0^\infty dT_0 \frac{x_0}{\sqrt{T_0^3}} \exp\left[-\frac{x_0^2}{2T_0}\right] \frac{1}{\sqrt{(\tau + T_0^{1/\beta})^\beta - T_0}} \propto \begin{cases} \frac{\Delta x}{\sqrt{\tau} x_0^{1-1/\beta}} & \text{for } \tau \ll x_0^{2/\beta}, \\ \frac{\Delta x}{\tau^{\beta/2}} & \text{for } \tau \gg x_0^{2/\beta}, \end{cases} \tag{S26}$$

in full agreement with the central result of this work, Eq. (1) of the main text.

B. Systematic numerical check of the scale-invariance of the time increments

Based on the RW models described above, we confirm the scale invariance of the following random variables :

- *The number of records.* We show in Supplementary Figure 2 that the number of records at time t is indeed scale invariant at large times as its average and standard deviation grow as expected as $t^{1/d_{\rm w}}$ for all non-Markovian process considered.
- *The time-increments between records.* Supplementary Figure 3 provides the distributions of the time increments $T_{m+n} - T_m$ and checks their scale invariance with respect to m and n; namely, that $(T_{m+n} - T_m)/n^{d^0_{\rm w}} m^{d_{\rm w} - d^0_{\rm w}}$ is independent of m and n for $n \ll m$.

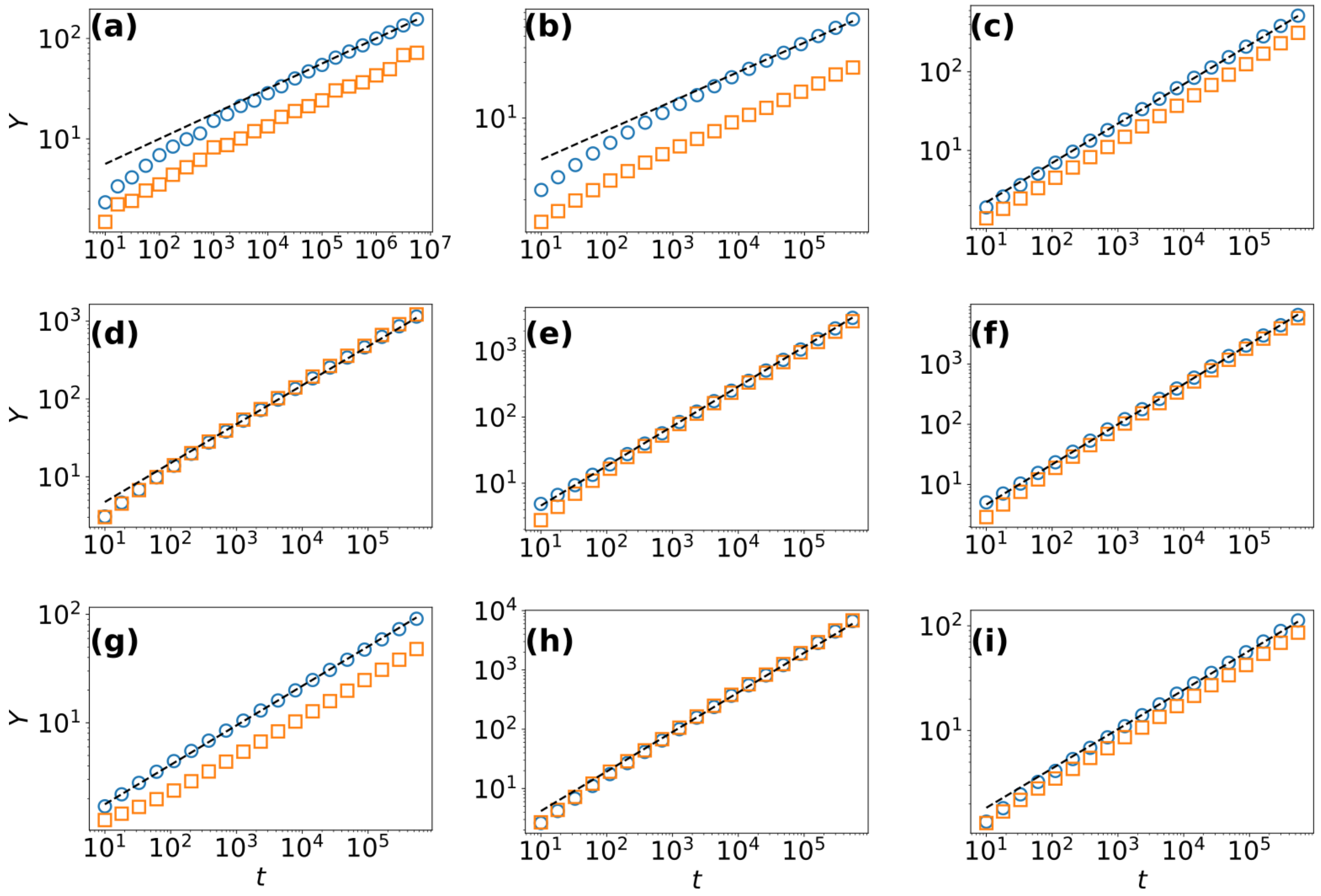

Supplementary Figure 2. **Average and standard deviation of number of records.** Each subpanel represents the average (blue circles) and standard deviation (orange squares) of the number of records reached at time t compared to the scaling expectation $\propto t^{1/d_{\rm w}}$ (dashed lines) for **(a)** fractional Brownian motion (fBm) of Hurst exponent $H = 0.25 = 1/d_{\rm w}$, **(b)** quenched fBm (qfBm) of Hurst exponent $H = 0.25 = 1/d_{\rm w}$, **(c)** elephant RW (eRW) of parameter $\beta = 0.25$ such that $d_{\rm w} = 2$, **(d)** Self-Attractive Walk (SATW) of parameter $\beta = 1$, such that $d_{\rm w} = 2$, **(e)** Sub-Exponential Self-Repelling Walk (SESRW) of parameter $\beta = 1$ and $\kappa = 0.5$ such that $d_{\rm w} = 5/3$, **(f)** True Self-Avoiding Walk (TSAW) of parameter $\beta = 1$ such that $d_{\rm w} = 3/2$, **(g)** Subdiffusive Average Lévy Lorentz (subALL) of parameter $a = 0.25$ such that $d_{\rm w} = 2.75$, $d^0_{\rm w} = 2$, **(h)** Superdiffusive Average Lévy Lorentz (supALL) of parameter $a = 0.5$ such that $d_{\rm w} = 3/2$, $d^0_{\rm w} = 2$, and **(i)** Scaled Brownian motion (sBm) of parameter $\beta = 0.75$ such that $d_{\rm w} = 8/3$.

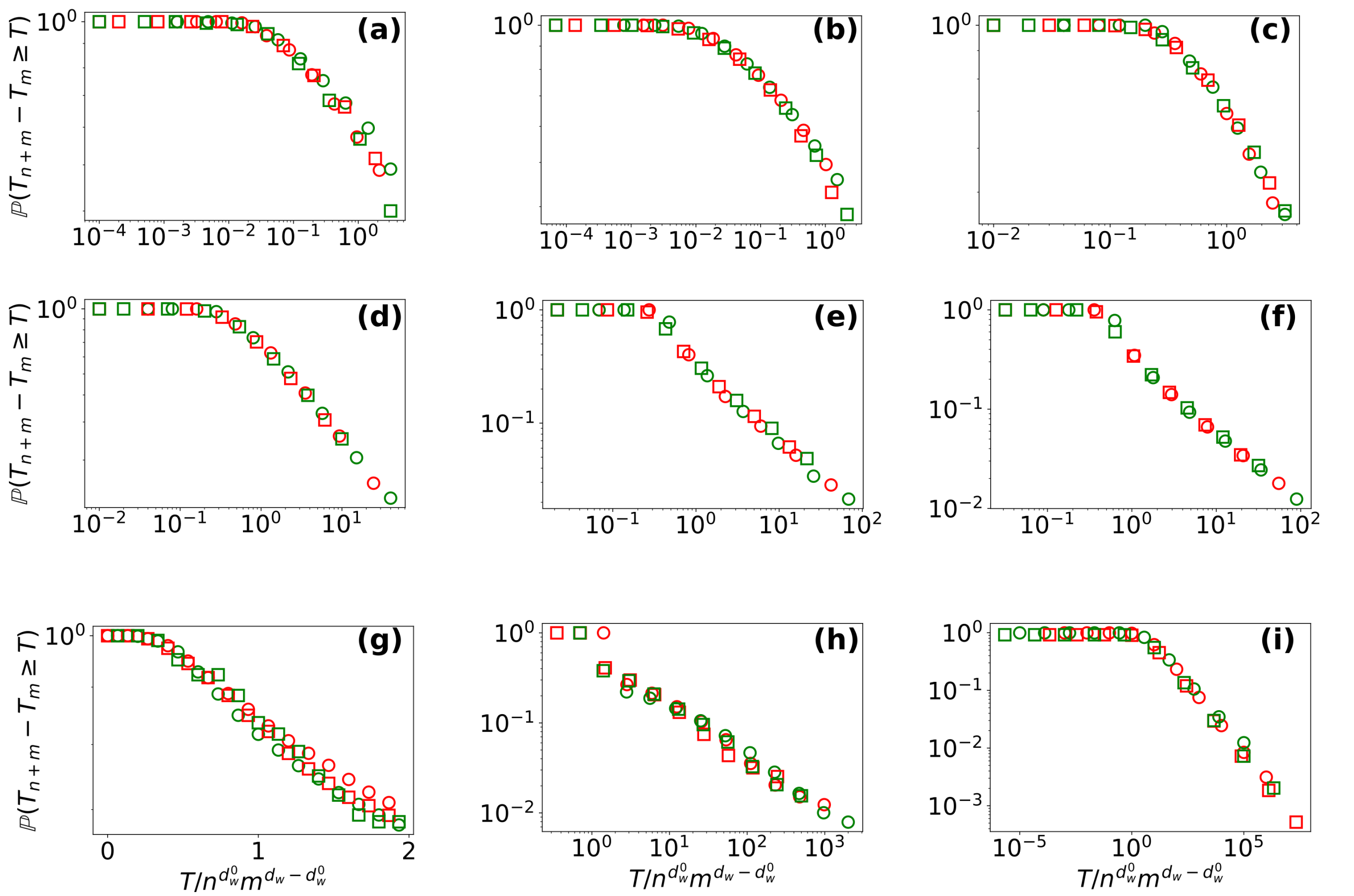

Supplementary Figure 3. **Scaled distribution of the time increments** $T_{m+n} - T_m$**.** Each subpanel represents the simulated tail distribution of the random variable $T_{m+n} - T_m$ as a function of $T/n^{d_\mathrm{w}^0} m^{d_\mathrm{w} - d_\mathrm{w}^0}$ for different values of n and m for **(a)** fBm of Hurst exponent $H = 0.25 = 1/d_\mathrm{w}$ ($n = 5$ and 10, $m = 50$ and 100) **(b)** qfBm of Hurst exponent $H = 0.25 = 1/d_\mathrm{w}$ ($n = 5$ and 10, $m = 50$ and 100) **(c)** eRW of parameter $\beta = 0.25$ such that $d_\mathrm{w} = 2$ ($n = 5$ and 10, $m = 50$ and 100) **(d)** SATW of parameter $\beta = 1$, such that $d_\mathrm{w} = 2$ ($n = 5$ and 10, $m = 100$ and 500) **(e)** SESRW of parameter $\beta = 1$ and $\kappa = 0.5$ such that $d_\mathrm{w} = 5/3$ ($n = 5$ and 10, $m = 100$ and 500) **(f)** TSAW of parameter $\beta = 1$ such that $d_\mathrm{w} = 3/2$ ($n = 5$ and 10, $m = 100$ and 500) **(g)** subALL of parameter $a = 0.25$ such that $d_\mathrm{w} = 2.75$, $d_\mathrm{w}^0 = 2$ ($n = 10$ and 20, $m = 400$ and 800) **(h)** supALL of parameter $a = 0.5$ such that $d_\mathrm{w} = 3/2$, $d_\mathrm{w}^0 = 2$ ($n = 5$ and 10, $m = 50$ and 100) **(i)** Exact tail distribution for sBm of parameter $\beta = 0.75$ such that $d_\mathrm{w} = 8/3$ ($n = 10$ and 100, $m = 1000$ and 10000). Increasing values of m are represented respectively by red and green symbols, while increasing values of n are represented respectively by circles and squares. Times $T_m > 10^6$ are discarded to have finite computation times.

C. Systematic numerical check of the asymptotic independence of record ages

Based on the RW models described above, we make systematic tests of the independence hypothesis between record ages:

- In Supplementary Figure 4, we check the effective independence hypothesis used in Eq. (3) of the main text, by comparing $\mathbb{P}(\max(\tau_m, \ldots, \tau_{m+n-1}) \leq T)$ with $\prod_{k=m}^{n+m-1}(1 - S(k,T))$ for various values of n in the regime n^{d_w} and T small in comparison to m^{d_w}. The functional forms being the same for both distributions even for different values of n (by rescaling with n and m), this confirms numerically the approximation for all the non-Markovian models considered.
- In Supplementary Figure 5, we display the probability to make at least δ successive records (event called a record run of length δ) when n record runs have been performed. In other words, we look at the joint distribution $\{\tau_n = 1, \ldots, \tau_{n+k} = 1\}$, which shows an exponential decay in the correlations between successive record ages, and thus provides an additional numerical check of the independence of τ_k. We note that the time decay rate of the exponential varies with n for aging RWs, as expected from the dependency on the number n of records of the early time regime for the record age τ_n.

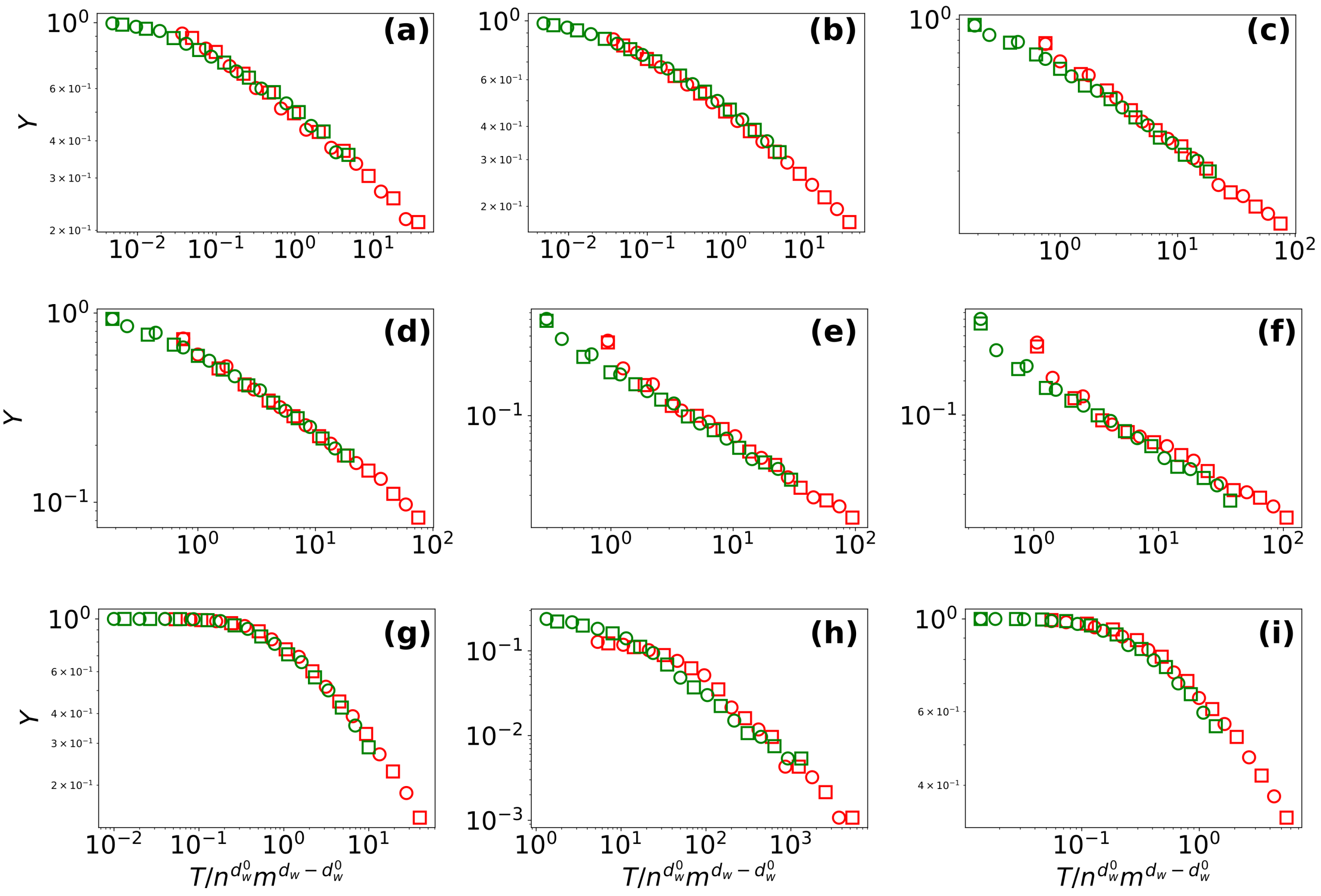

Supplementary Figure 4. **Scaled distribution of the record maximum with and without the independence approximation.** Each subpanel represents the tail distribution of the random variable $\max(\tau_m, \ldots, \tau_{n+m-1})$, $\mathbb{P}(\tau_k > T,\ k = m, \ldots, n+m-1) = 1 - \mathbb{P}(\tau_k \leq T,\ k = m, \ldots, n+m-1)$ (circles), and the product tail distribution of $\tau_m, \ldots, \tau_{n+m-1}$, $1 - \prod_{k=m}^{m+n-1} \mathbb{P}(\tau_k \leq T)$ (squares), as a function of $T/n^{d_\mathrm{w}^0} m^{d_\mathrm{w} - d_\mathrm{w}^0}$ with $n = 2$ (red), $n = 4$ (green) and $m = 50$ for **(a)** fBm of Hurst exponent $H = 0.25 = 1/d_\mathrm{w}$, **(b)** qfBm of Hurst exponent $H = 0.25 = 1/d_\mathrm{w}$ ($n = 2$ and 4), **(c)** eRW of parameter $\beta = 0.25$ such that $d_\mathrm{w} = 2$, **(d)** SATW of parameter $\beta = 1$, such that $d_\mathrm{w} = 2$, **(e)** SESRW of parameter $\beta = 1$ and $\kappa = 0.5$ such that $d_\mathrm{w} = 5/3$, **(f)** TSAW of parameter $\beta = 1$ such that $d_\mathrm{w} = 3/2$, **(g)** SubALL of parameter $a = 0.25$ such that $d_\mathrm{w} = 2.75$, $d_\mathrm{w}^0 = 2$, **(h)** SupALL of parameter $a = 0.5$ such that $d_\mathrm{w} = 3/2$, $d_\mathrm{w}^0 = 2$, and **(i)** sBm of parameter $\beta = 0.75$ such that $d_\mathrm{w} = 8/3$. Times $T_{m+n} > 10^6$ are discarded to have finite computation times.

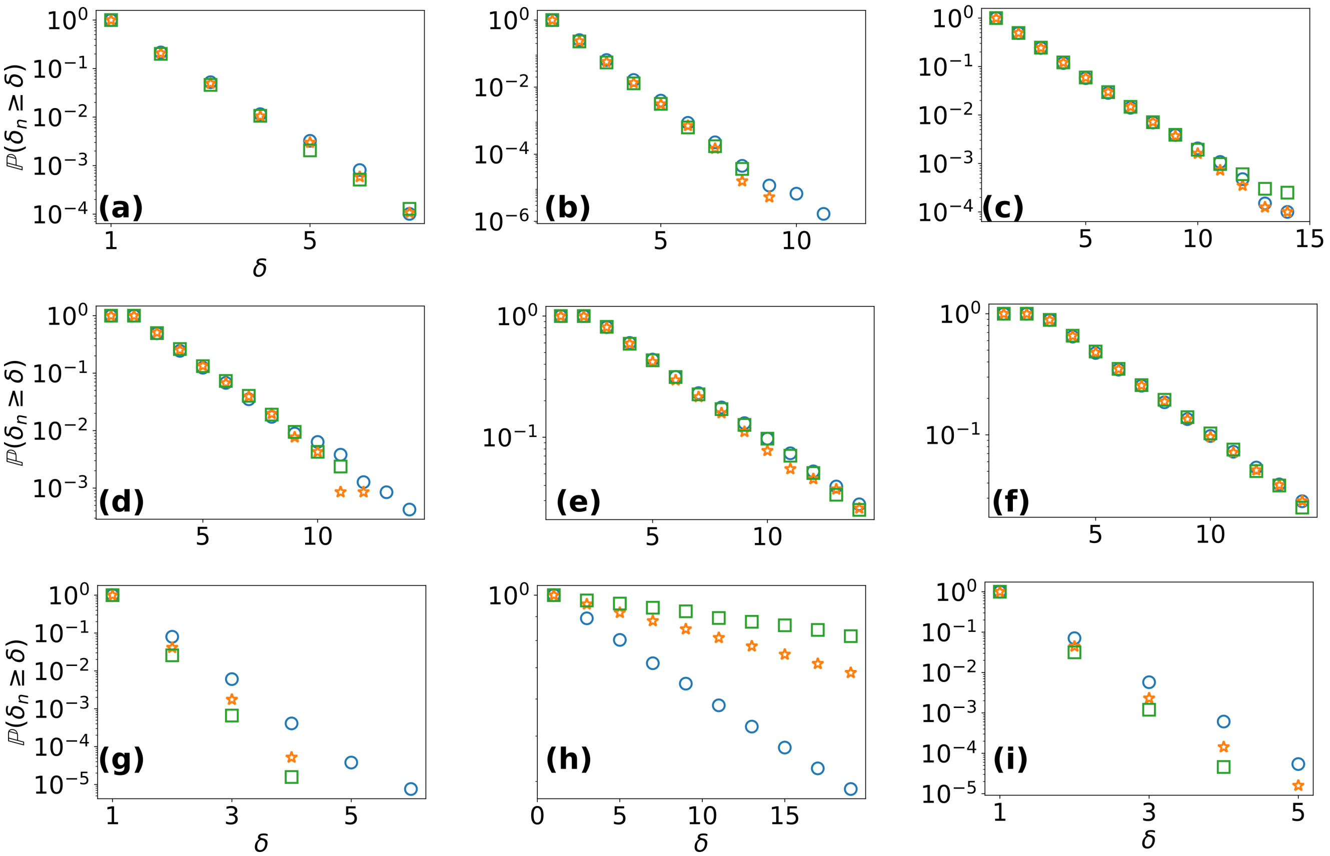

Supplementary Figure 5. **Distributions of record runs.** Each subpanel represents the distribution of a record run δ_n for different values of the number n of previous record runs for **(a)** fBm of Hurst exponent $H = 0.25 = 1/d_{\mathrm{w}}$, **(b)** qfBm of Hurst exponent $H = 0.25 = 1/d_{\mathrm{w}}$, **(c)** eRW of parameter $\beta = 0.25$ such that $d_{\mathrm{w}} = 2$, **(d)** SATW of parameter $\beta = 1$, such that $d_{\mathrm{w}} = 2$, **(e)** SESRW of parameter $\beta = 1$ and $\kappa = 0.5$ such that $d_{\mathrm{w}} = 5/3$, **(f)** TSAW of parameter $\beta = 1$ such that $d_{\mathrm{w}} = 3/2$, **(g)** subALL of parameter $a = 0.25$ such that $d_{\mathrm{w}} = 2.75$, $d_{\mathrm{w}}^0 = 2$, **(h)** supALL of parameter $a = 0.5$ such that $d_{\mathrm{w}} = 3/2$, $d_{\mathrm{w}}^0 = 2$, and **(i)** sBm of parameter $\beta = 0.75$ such that $d_{\mathrm{w}} = 8/3$. All distributions are computed for $n = 10$, 25 and 50 record runs (blue circles, orange stars and green squares). Times to reach n record runs larger than 10^6 are discarded to have finite computation times.

S3. DATA ANALYSIS

We provide a comprehensive description of the datasets used in this study, along with the general methodology that yields the results presented in the main text. Furthermore, we include complementary datasets that provide additional confirmation of our findings, including cases involving aging time series.

A. Details on the datasets used in the main text

Here, we present the datasets used in the main text:

(a) *Elbe river discharge (m^3/s).* We consider the daily mean debit of the Elbe river measured in Dresden [S27]. It was observed that this quantity presents correlation which can be modeled by subdiffusive non-Markovian RWs [S28]. In this time series, we obtain $H \approx 0.14$ by application of the DMA method [S29, S30], see below.

(b) *Volcanic soil temperature (^oC).* It was shown in [S31, S32] that the soil temperature monitored in the volcanic caldera of the Campi Flegrei area in Naples follows an fBm of parameter $H \approx 0.42$ once the data have been detrended by removing the linear trends between two temperature extrema (between two solstices). Here we also detrend the data by removing the same linear seasonal trends. We display the data measured at the Monte Olibano (OLB) site.

(c) *Trajectories of microspheres in agarose gel* (nm). The trajectories [S33] represent the $2d$ motion of 50-nm polystyrene microspheres in agarose hydrogel (we consider that x and y displacement are i.i.d., giving us 2 independent $1d$ trajectories). There are 20 trajectories of 2000 frames which were analyzed in [S33] who obtained $1/d_{\mathrm{w}} \approx 0.43$.

(d) *Motion of amoeba intracellular vacuoles* ($pixels = 106nm$). We consider vacuole intracellular trajectories inside the amoeba in a $2d$ plane (we consider that x and y displacement are i.i.d., giving us 2 independent $1d$ trajectories) of at least 2048 frames. It was estimated in [S33] that the walk dimension verifies $1/d_{\mathrm{w}} \approx 0.67$.

(e) *Trajectories of telomeres (μm).* We use $2d$ trajectories [S33] of telomeres in the nucleus of untreated U2OS cells obtained in Ref. [S34] (we consider that x and y displacement are i.i.d., giving us 2 independent $1d$ trajectories). Similarly to Ref. [S33], we only consider trajectories where the mean-square displacement grows as $t^{0.5\pm0.05}$, which corresponds to $1/d_{\mathrm{w}} \approx 0.25$.

(f) *DNA RW on the Homo sapiens β-myosin heavy chain (HUMBMYH7).* It was observed in [S35, S36] that the process is a RW with long-range correlations of Hurst exponent $H \approx 0.67$. We estimate and remove the bias $\hat{v} = \frac{1}{N}\sum_{t=1}^{N}\eta_t$ in the data by replacing η_t by $\eta_t - \hat{v}$.

(g) *Cumulative London air temperature* ($^oC.day$). In this case, the temperature fluctuations (as for **(b)**, we remove the linear trends between two solstices) are fractional Gaussian noise (fGn), in agreement to what was observed in [S37], of Hurst exponent $H \approx 0.8$ obtained via the DMA method [S29, S30], see below. We consider the cumulative temperature fluctuation, which is then fBm. This quantity is of a particular interest in the studies of derivative pricing (see [S37]).

(h) *Cumulative Ethernet traffic* (10 *bytes.ms*). The dataset represents the number of packets going through an Ethernet cable every 10 *ms* at the Bellcore Morristown Research and Engineering facility [S38]. In particular, in [S39], it was shown that the process is a fGn of dimension $H \approx 0.8$. As for **(e)**, the cumulative number of requests up to a given time t gives a non-Markovian process. We detrend the cumulative data as for **(g)** by removing the estimated bias $\hat{v}$ at every step. We display the measurement performed in August 1989.

B. Characterization and parametrization of the data used in the main text

In this section we provide the method developed to determine the walk dimension of the time series presented in the main text as well as numerical checks of their stationarity.

In order to obtain the walk dimension d_{w} in a time series, one applies the celebrated Detrending Moving Average (DMA) [S29, S30] method, which consists in evaluating the typical fluctuations in a window of size ℓ regardless of any bias or deterministic trend. More precisely, for a dataset $(X_t)_{t=0,\dots,N}$, we consider the windows of size

up to $\ell_{\max}$, compute the window averages $x_t^\ell = \frac{1}{\ell}\sum_{i=0}^{\ell-1} X_{t-i}$, and the typical fluctuation for a window of size ℓ, $F(\ell) = \sqrt{\frac{1}{N-\ell_{\max}}\sum_{t=\ell_{\max}}^{N}(X_t - x_t^\ell)^2}$. When several trajectories are available, we consider the average fluctuation over all the trajectories (for telomeres, vacuoles and microspheres in agarose data). If the data behave as a RW of walk dimension d_{w}, then $F(\ell) \propto \ell^{1/d_{\mathrm{w}}}$. We obtain the value of $1/d_{\mathrm{w}}$ via the DMA method (first two lines in Supplementary Figure 6). Then, we compare the exponent with that obtained from the Mean Square Displacement (MSD). As one can see in the last two lines of Supplementary Figure 6, the MSD has the algebraic growth predicted by the DMA method, which indicates that the deterministic trends have been removed correctly.

In order to check that the data are stationary, we compare the MSD obtained from the increments $\{x_t = X_{t+T} - X_T\}_{T\leq N/4,t}$ in the first quarter of the data and the increments $\{x_t = X_{t+T} - X_T\}_{3N/4\leq T,t}$ in the last quarter of the data. Indeed, for all datasets the MSD in the two sub-intervals of the data have similar growth, i.e. the aging exponent is $\alpha = 0$. We note that for the river flow dataset there is difference a constant prefactor. This transient aging explains the small deviations in Fig. 3 **(a')** of the main text from the behavior of a stationary process characterized by the persistence exponent $\theta = 1 - 1/d_{\mathrm{w}}$. However, since the walk dimension is not changed, $\alpha = 0$ and the record age exponent is still $1/d_{\mathrm{w}}$ for this dataset.

Record ages are obtained by starting the subtrajectories at values of t equally spaced at intervals at least 200 time steps long, and observing successive records occurring in the subtrajectory. First return times are obtained by starting the subtrajectories at any value of time.

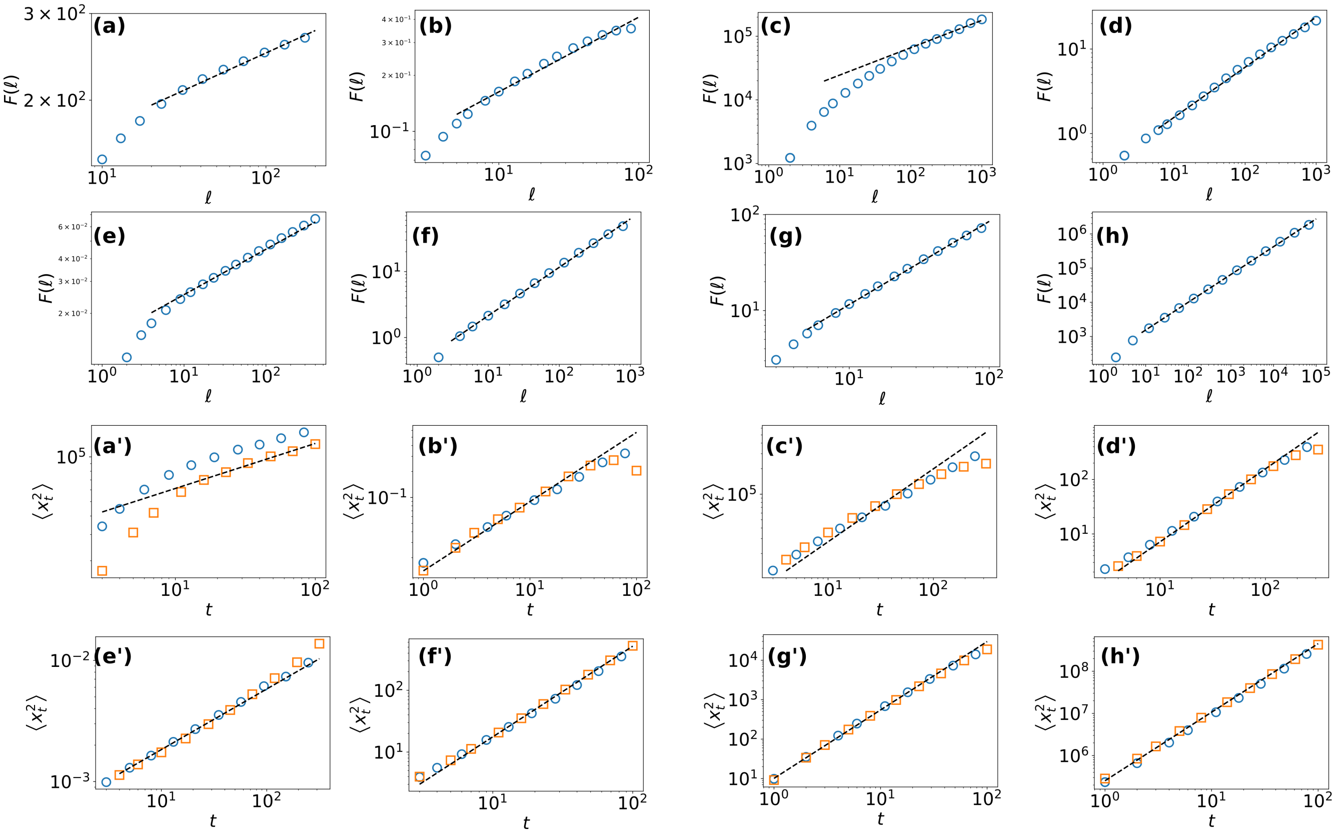

Supplementary Figure 6. **Characterization of the data used in Fig. 3 of the main text:**
(a) river discharge, **(b)** volcanic soil temperature, **(c)** motion of microspheres in a gel, **(d)** motion of vacuoles inside an amoeba, **(e)** motion of telomeres, **(f)** DNA RW, **(g)** cumulative air temperature, and **(h)** Ethernet cumulative requests.
Top subfigures **(a)**- **(h)** show $F(\ell)$ obtained via the DMA method. The linear fit on the log-log plot is represented by a black dashed line.
Bottom subfugures **(a′)**- **(h′)** show the MSD $\langle x_t^2\rangle$ computed from the first (blue circles) and last (orange squares) quarters of the data. Black dashed line stands for the algebraic growth $t^{2/d_{\mathrm{w}}}$ where d_{w} was obtained via the DMA method.

C. Analysis of complementary datasets

We additionally conducted an analysis of the following complementary datasets to further demonstrate the broad applicability of our results:

(**a**) *Volcanic soil temperature* (^oC). Another dataset (measured at the Monte Sant'Angelo site) of soil temperatures monitored in the volcanic caldera of the Campi Flegrei area in Naples. It was shown in [S31, S32] that it is an fBm of parameter $H \approx 0.4$ once the data have been detrended by removing the linear trends between two temperature extrema (between two solstices). Here we also detrend the data by removing the same linear seasonal trends.

(**b**) *Cumulative air temperature at Montélimar* ($^oC.day$). As for the soil temperature in [S31], we remove the linear trends between two solstices. In this case, the temperature fluctuations are fractional Gaussian noise (fGn) and not fBm, in agreement to what was observed in [S37] for the data at London. We obtain a Hurst exponent $H \approx 0.8$ via the DMA method.

(**c**) *Rhône river discharge* (m^3/s). We consider the daily mean debit of the Rhône measured at the Sault Brenaz station [S40]. It was observed that this quantity presents correlations which can be modeled by subdiffusive non-Markovian RW [S28]. In this time series, we obtain $H \approx 0.21$ by applications of the DMA method.

(**d**) *DNA RW.* We consider the human T-cell receptor α/δ sequence from the GenBank data base (HUMTCRADCV). It was observed in [S35, S36] that the process is a RW with long-range correlations of Hurst exponent $H \approx 0.61$. We estimate and remove the bias $\hat{v} = \frac{1}{N}\sum_{t=1}^{N} \eta_t$ in the data by replacing η_t by $\eta_t - \hat{v}$.

(**e**) *Cumulative Ethernet traffic* (10 *bytes.ms*). The dataset represents the number of packets going through an Ethernet cable every 10 *ms* at the Bellcore Morristown Research and Engineering facility [S38]. In particular, in [S39], it was shown that the process is a fGn of dimension $H \approx 0.84$. This is why we consider the cumulative number of requests up to a given time t. We detrend the cumulative data in the same manner as the DNA RW by removing the estimated bias $\hat{v}$ at every step. We display the data measured in October 1989.

All considered complementary datasets support our theoretical results.

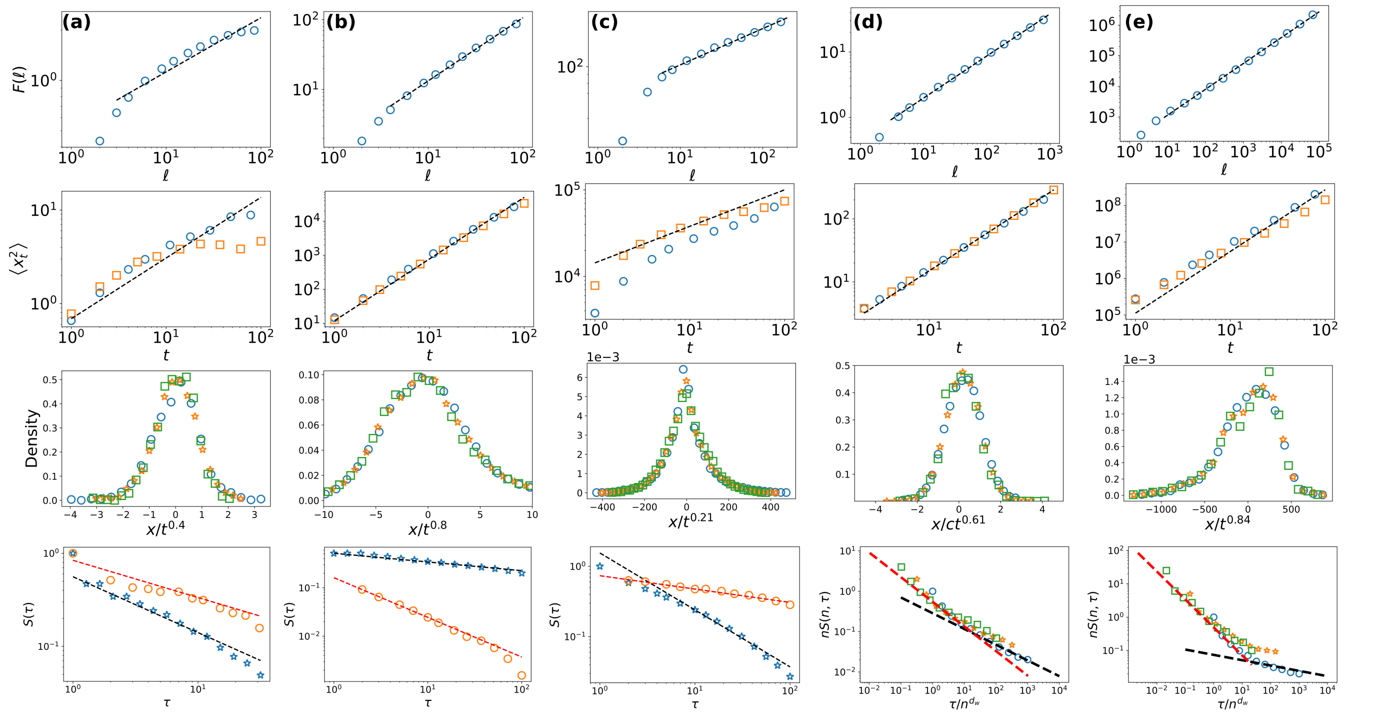

Supplementary Figure 7. **Analysis of record ages for non-Markovian RWs: theoretical predictions (lines) vs real time observations (symbols): (a)** volcanic soil temperature (Monte Sant'Angelo site), **(b)** cumulative air temperature (Montélimar), **(c)** river discharge (Rhône), **(d)** DNA RW (HUMTCRADCV), and **(e)** Ethernet cumulative requests (October 1989).
First line, $F(\ell)$ obtained via the DMA method and linear fit to the log-log representation shown by black dashed line.
Second line, check of the MSD $\langle x_t^2 \rangle$ computed from the first (blue circles) and last (orange squares) quarters of the data. Black dashed line shows linear fit to the log-log representation of the MSD data, given the same exponent $2/d_{\rm w}$.
Third line, distribution of the increment $x_t = X_{t+T} - X_T$ at different times t normalised by $t^{1/d_{\rm w}}$, where $d_{\rm w}$ was obtained via the DMA method for: **(a)** $t = 5$, 10 and 20 **(b)** $t = 5$, 10 and 20 **(c)** $t = 10$, 20 and 40 **(d)** $t = 20$, 40 and 80 **(e)** $t = 500$, 1000 and 2000. Increasing values of times are represented successively by blue circles, orange stars and green squares.
Fourth line, **(a)**-**(c)** statistics of the time to first reach the initial value in the sub interval (blue stars) and the statistics of the records (regardless of the number n of records, orange circles) and **(d)**-**(e)** rescaled tail distribution of record ages τ_n for different values of the number of records n ($n = 1$, 2 and 4 for **(d)** and $n = 1$, 5, 25 for **(e)**). The black dashed line represents the algebraic decay $\tau^{-\theta}$ while the red dashed line stands for the algebraic decay $\tau^{-1/d_{\rm w}}$.

D. Datasets displaying aging of the increments

We also analysed the following complementary datasets which present aging in the increments:

(a) *Single cell displacement on a* $1d$ *medium (half-pixels=0.65μm).* We analyze the motion of MDCK (Madin-Darby Canine Kidney) epithelial cells on micro-contact-printed $1d$ linear tracks of fibronectin obtained in [S41], who found that the cells perform a Persistent Self-Attractive Walk (PSATW, which is a generalization of the SATW with a finite correlation length) motility behaviour, such that $d_{\rm w} = d_{\rm w}^0 = 2$. Because of the (transient, $\alpha = 0$) aging in the data, the persistence exponent θ is different from 1/2.

(b) *Single cell displacement on a* $2d$ *medium (pixels=1.3μm).* We analyze the motion of MDCK epithelial cells on a $2d$ substrate obtained in [S41], who found that the cells show a PSATW motility behaviour, such that $d_{\rm w} = 3$ and $d_{\rm w}^0 = 2$ in agreement with theoretical and numerical observations for this type of model in $2d$ [S42, S43]. We consider that motion in x and y are i.i.d. and thus form two independent $1d$ trajectories. Because of the aging in the data, the persistence exponent θ is different from 1/2.

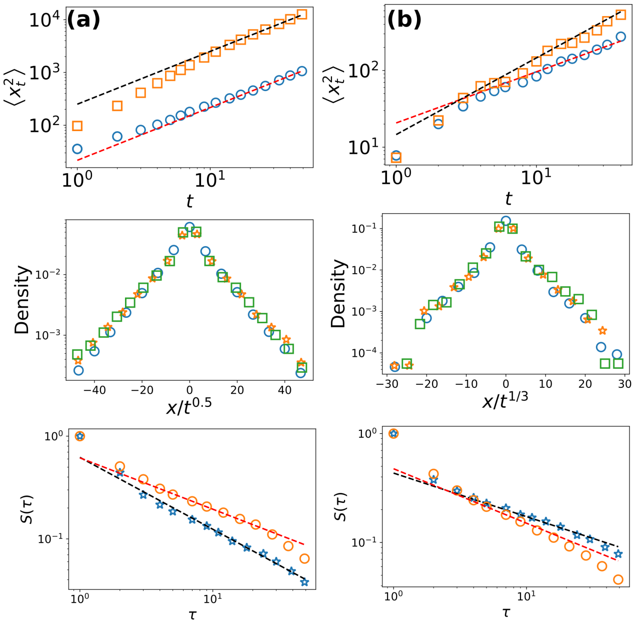

Supplementary Figure 8. **Analysis of record ages for non-Markovian RWs: theoretical predictions (lines) vs experimental data displaying aging of the increments (symbols).** Cell motility on a **(a)** $1d$ and **(b)** $2d$ micropatterned surfaces.
First line, MSD $\langle x_t^2 \rangle$ computed at early times (blue circles, to compare with the red line $\propto t^{2/d_{\rm w}}$) and at latter times (orange squares, to compare with the black line $\propto t^{2/d_{\rm w}^0}$).
Second line, distribution of the increment $x_t = X_{t+T} - X_T$ at different times t normalised by $t^{1/d_{\rm w}}$, where $d_{\rm w}$ was derived in [S41], for $t = 5$, 10 and 20 ($T < 50$). Increasing values of times are represented successively by blue circles, orange stars and green squares.
Third line, statistics of the time to first reach the initial value in the sub interval (blue stars) and the statistics of the records (regardless of the number n of records, orange circles). The black dashed line stands for the algebraic decay $\tau^{-\theta}$, where θ is estimated to be **(a)** $\theta \approx 0.7$ and **(b)** $\theta \approx 0.4$, and the red line represents the algebraic decay $\tau^{-1/d_{\rm w}^0}$.

Supplementary Figure 8 shows the analysis of datasets **(a)** and **(b)**. Note that, because of aging, the persistence exponent $\theta \neq 1 - 1/d_{\rm w}$. We conclude that these complex examples also support our theory.

SUPPLEMENTARY REFERENCES

[S1] J. H. P. Schulz, E. Barkai, and R. Metzler, Aging renewal theory and application to random walks, Phys. Rev. X **4**, 011028 (2014).
[S2] N. Levernier, O. Bénichou, T. Guérin, and R. Voituriez, Universal first-passage statistics in aging media, Phys. Rev. E **98**, 022125 (2018).
[S3] C. Godrèche and J.-M. Luck, Record statistics of integrated random walks and the random acceleration process, J. Stat. Phys. **186**, 4 (2022).
[S4] J.-P. Bouchaud and A. Georges, Anomalous diffusion in disordered media: Statistical mechanisms, models and physical applications, Phys. Rep. **195**, 127–293 (1990).
[S5] D. Carpentier and P. Le Doussal, Glass transition of a particle in a random potential, front selection in nonlinear renormalization group, and entropic phenomena in Liouville and sinh-Gordon models, Phys. Rev. E **63**, 026110 (2001).
[S6] A. J. Bray, S. N. Majumdar, and G. Schehr, Persistence and first-passage properties in nonequilibrium systems, Adv. Phys. **62**, 225 (2013).
[S7] J. Krug, H. Kallabis, S. Majumdar, S. Cornell, A. J. Bray, and C. Sire, Persistence exponents for fluctuating interfaces, Phys. Rev. E **56**, 2702 (1997).
[S8] A. Hansen, T. Engøy, and K. J. Måløy, Measuring hurst exponents with the first return method, Fractals **2**, 527 (1994).
[S9] M. Ding and W. Yang, Distribution of the first return time in fractional brownian motion and its application to the study of on-off intermittency, Phys. Rev. E **52**, 207 (1995).
[S10] S. Maslov, M. Paczuski, and P. Bak, Avalanches and $\frac{1}{f}$ noise in evolution and growth models, Phys. Rev. Lett. **73**, 2162 (1994).
[S11] C. S. Ryu and I.-m. Kim, Solid-on-solid model with next-nearest-neighbor interaction for epitaxial growth, Phys. Rev. E **52**, 2424 (1995).
[S12] A. Barbier-Chebbah, O. Benichou, and R. Voituriez, Anomalous persistence exponents for normal yet aging diffusion, Phys. Rev. E **102**, 062115 (2020).
[S13] V. B. Sapozhnikov, Self-attracting walk with $\nu < 1/2$, J. Phys. A: Math. Gen. **27**, L151 (1994).
[S14] B. Davis, Reinforced random walk, Probab. Theor. Rel. Fields **84**, 203–229 (1990).
[S15] A. Barbier-Chebbah, O. Bénichou, and R. Voituriez, Self-interacting random walks: Aging, exploration, and first-passage times, Phys. Rev. X **12**, 011052 (2022).
[S16] H. C. Ottinger, The generalised true self-avoiding walk-a model with continuously variable exponent ν, J. Phys. A: Math. Gen. **18**, L363 (1985).
[S17] D. J. Amit, G. Parisi, and L. Peliti, Asymptotic behavior of the "true" self-avoiding walk, Phys. Rev. B **27**, 1635 (1983).
[S18] L. Pietronero, Critical dimensionality and exponent of the "true" self-avoiding walk, Phys. Rev. B **27**, 5887 (1983).
[S19] S. P. Obukhov and L. Peliti, Renormalisation of the 'true' self-avoiding walk, J. Phys. A: Math. Gen. **16**, L147 (1983).
[S20] B. Toth, The "True" Self-Avoiding Walk with Bond Repulsion on $\mathbb{Z}$: Limit Theorems, Ann. Probab. **23**, 1523 (1995).
[S21] M. Radice, M. Onofri, R. Artuso, and G. Cristadoro, Transport properties and ageing for the averaged lévy–lorentz gas, J. Phys. A: Math. Theor. **53**, 025701 (2019).
[S22] M. Radice, M. Onofri, R. Artuso, and G. Pozzoli, Statistics of occupation times and connection to local properties of nonhomogeneous random walks, Phys. Rev. E **101**, 042103 (2020).
[S23] H. Safdari, A. V. Chechkin, G. R. Jafari, and R. Metzler, Aging scaled brownian motion, Phys. Rev. E **91**, 042107 (2015).
[S24] S. C. Lim and S. V. Muniandy, Self-similar gaussian processes for modeling anomalous diffusion, Phys. Rev. E **66**, 021114 (2002).
[S25] J.-H. Jeon, A. V. Chechkin, and R. Metzler, Scaled brownian motion: a paradoxical process with a time dependent diffusivity for the description of anomalous diffusion, Phys. Chem. Chem. Phys. **16**, 15811 (2014).
[S26] Y. He, S. Burov, R. Metzler, and E. Barkai, Random time-scale invariant diffusion and transport coefficients, Phys. Rev. Lett. **101**, 058101 (2008).
[S27] GRDC, Dresden mean daily discharge (1920-1992), data from the Global Runoff Data Centre (GRDC), `https://portal.grdc.bafg.de/applications/`.
[S28] Q. Zhang, C.-Y. Xu, Y. D. Chen, and Z. Yu, Multifractal detrended fluctuation analysis of streamflow series of the Yangtze river basin, China, Hydrol. Process. **22**, 4997 (2008).
[S29] M. Höll, K. Kiyono, and H. Kantz, Theoretical foundation of detrending methods for fluctuation analysis such as detrended fluctuation analysis and detrending moving average, Phys. Rev. E **99**, 033305 (2019).
[S30] E. Alessio, A. Carbone, G. Castelli, and V. Frappietro, Second-order moving average and scaling of stochastic time series, Eur. Phys. J. B **27**, 197 (2002).
[S31] A. Di Crescenzo, B. Martinucci, and V. Mustaro, A model based on fractional brownian motion for temperature fluctuation in the Campi Flegrei caldera, Fractal Fract. **6**, 421 (2022).
[S32] C. Sabbarese, F. Ambrosino, G. Chiodini, F. Giudicepietro, G. Macedonio, S. Caliro, W. De Cesare, F. Bianco, M. Pugliese, and V. Roca, Continuous radon monitoring during seven years of volcanic unrest at Campi Flegrei caldera (Italy), Sci. Rep. **10**, 9551 (2020).
[S33] D. Krapf, N. Lukat, E. Marinari, R. Metzler, G. Oshanin, C. Selhuber-Unkel, A. Squarcini, L. Stadler, M. Weiss, and X. Xu, Spectral content of a single non-brownian trajectory, Phys. Rev. X **9**, 011019 (2019).
[S34] L. Stadler and M. Weiss, Non-equilibrium forces drive the anomalous diffusion of telomeres in the nucleus of mammalian cells, New J. Phys. **19**, 113048 (2017).

[S35] C.-K. Peng, S. V. Buldyrev, S. Havlin, M. Simons, H. E. Stanley, and A. L. Goldberger, Mosaic organization of dna nucleotides, Phys. Rev. E **49**, 1685 (1994).
[S36] C.-K. Peng, S. V. Buldyrev, A. L. Goldberger, S. Havlin, F. Sciortino, M. Simons, and H. E. Stanley, Long-range correlations in nucleotide sequences, Nature **356**, 168 (1992).
[S37] D. C. Brody, J. Syroka, and M. Zervos, Dynamical pricing of weather derivatives, Quant. Finance **2**, 189 (2002).
[S38] H. Fowler and W. Leland, Local area network characteristics, with implications for broadband network congestion management, IEEE J. Sel. Areas Commun. **9**, 1139 (1991).
[S39] W. E. Leland, M. S. Taqqu, W. Willinger, and D. V. Wilson, On the self-similar nature of ethernet traffic, in *Conference proceedings on Communications architectures, protocols and applications* (1993) pp. 183–193.
[S40] GRDC, Rhône mean daily discharge at the sault brenaz station (1920-1992), data from the Global Runoff Data Centre (GRDC), `https://portal.grdc.bafg.de/applications/`.
[S41] J. d'Alessandro, A. Barbier-Chebbah, V. Cellerin, O. Benichou, R. M. Mège, R. Voituriez, and B. Ladoux, Cell migration guided by long-lived spatial memory, Nat. Commun. **12**, 4118 (2021).
[S42] A. Ordemann, E. Tomer, G. Berkolaiko, S. Havlin, and A. Bunde, Structural properties of self-attracting walks, Phys. Rev. E **64**, 046117 (2001).
[S43] J. G. Foster, P. Grassberger, and M. Paczuski, Reinforced walks in two and three dimensions, New J. Phys. **11**, 023009 (2009).
